# Insights into the epidemiology, risk factors, and clinical outcomes of carbapenem-resistant *Acinetobacter baumannii* infections in critically ill children

**DOI:** 10.3389/fpubh.2023.1282413

**Published:** 2023-11-30

**Authors:** Yufei Zhang, Guifeng Xu, Fei Miao, Weichun Huang, Haiying Wang, Xing Wang

**Affiliations:** ^1^Department of Clinical Laboratory, Yueyang Hospital of Integrated Traditional Chinese and Western Medicine, Shanghai University of Traditional Chinese Medicine, Shanghai, China; ^2^Innovation Research Institute of Traditional Chinese Medicine, Shanghai University of Traditional Chinese Medicine, Shanghai, China; ^3^Department of Dermatology, Huadong Hospital, Fudan University, Shanghai, China; ^4^Department of Laboratory Medicine, Shanghai Children's Medical Center, Shanghai Jiaotong University School of Medicine, Shanghai, China

**Keywords:** carbapenem-resistant *Acinetobacter baumannii*, epidemiology, pediatric, risk factor, mortality

## Abstract

**Background and aims:**

Carbapenem-resistant *Acinetobacter baumannii* (CRAB) has become a leading cause of nosocomial infections with an increasing impact on critically ill patients, yet there is limited data on contributing factors. This study was aim to evaluate the prevalence and risk factors, and clinical outcomes of CRAB infections among critically ill children in a tertiary university teaching hospital in China.

**Methods:**

From January 2016 to December 2021, all children diagnosed with nosocomial *Acinetobacter baumannii* (*A. baumannii*) infections in the pediatric intensive care unit (PICU) were identified through the computerized microbiology laboratory databases. Among them, children suffering from CRAB infection were designated as a case group, while children with carbapenem susceptible *A. baumannii* (CSAB) infection were assigned to a control group. This retrospective case-control study was based on two groups of patients to determine potential clinical factors contributing to CRAB infection and death among critically ill children via univariate and multivariate analyses.

**Results:**

During the 6-year study period, a total of 372 episodes of nosocomial *A. baumannii* infection in the PICU were eligible and included in the study. These isolates displayed moderate or high rates of resistance to all tested antimicrobials except colistin. The overall prevalence of CRAB and MDRAB (multidrug-resistant *A. baumannii*) was 78.0% and 80.9%, respectively. Several risk factors found to significantly increase CRAB infection included receiving invasive operation (OR = 9.412, *p* = 0.001), gastric intubation (OR = 2.478, *p* = 0.026), prior carbapenems exposure (OR = 2.543, *p* = 0.003), severe pneumonia (OR = 3.235, *p* = 0.001), and hemoglobin <110g/L (OR = 3.049, *p* = 0.005). Of 372 patients with CRAB infection, the mortality rate was 30.9% (115/372) and mortality did not differ between children with CRAB and CSAB infections. Septic shock (OR = 2.992, *p* = 0.001), AST > 46U/L (OR = 2.015, *p* = 0.005), bone marrow aspiration (OR = 2.704, *p* = 0.008), lymphocyte <20 % (OR = 1.992, *p* = 0.006) and age (OR = 1.094, *p* = 0.002) were independent risk factors for the death of *A. baumanni* infection.

**Conclusions:**

This study highlights considerable incidence rate and remarkable mortality of children with *A. baumanni* (especially CRAB) infections, and identifies age-specific risk factors for CRAB infection and mortality in critically ill children. These risk factors should be taken into account in pediatric hospitals in order to establish early intervention and rational treatment to improve clinical outcomes.

## Introduction

*Acinetobacter baumannii* is one of the most important nosocomial pathogens, with the ability to cause infections such as ventilator-associated pneumonia, surgical site infections, urinary tract infections, bloodstream infections and meningitis (1, 2). These infections are more likely to occur in intensive care unit (ICU) patients, although transmission to patients in general wards and long-term care facilities is increasing (3). A study conducted at one hospital in India found that *A. baumannii* accounted for 9.4% of all Gram-negative bacteria in the entire hospital and 22.6% in the ICU (4). According to the Infectious Disease Surveillance of Pediatrics (ISPED) program in China, *A. baumannii* was among the 10 most frequent etiologic agents and was the fourth leading pathogen responsible for traumatic infections in children (5). The European Antimicrobial Resistance Surveillance Network (EARS-Net) reported that 29 countries isolated 7622 *Acinetobacter* spp, constituting 2.3% of all isolates in 2020 (6). In the United States, a national surveillance report claims that *A. baumannii* was responsible for 1.8% of all healthcare-associated infections, with an estimated 45,000 *Acinetobacter* infections per year and 1 million cases per year worldwide (7).

*A. baumannii* has become a successful nosocomial pathogen worldwide due to the combination of its environmental adaptation and rapid development of resistance to multiple antimicrobials (8). Given its innate or acquired capacity for antibiotic resistance, carbapenem-resistant or multidrug-resistant (MDR) *A. baumannii* has substantially increased. In China, an antimicrobial resistance surveillance network indicated that the prevalence of CRAB showed a significantly increasing trend from 39.0% in 2005 to 71.9% in 2022 with a peak of 79.0% in 2019 (9). In the USA, the proportion of CRAB in children was on the rise, which increased each year by 8% from 1999 to 2012 (10). Compared with 2016-2019, the percentage of carbapenems resistant to *Acinetobacter spp*. in the European Union/European Economic Area (EU/EEA) increased significantly in 2020, which was higher than that of carbapenems resistant to *P. aeruginosa* or *K. pneumonia* (6).

The rapid increase and worldwide spread of CRAB infection have become a serious public health concern, as it is difficult to treat and associated with high mortality and morbidity. In 2019, the U.S. Centers for Disease Control (CDC) designated CRAB an urgent threat to public health, which caused approximately 8500 hospital-associated infections and 700 deaths in 2017 (7). It is important to note that CRAB infections can be disastrous for ICU patients. Among these patients, underlying diseases, immune deficiency and invasive operations all contributed to the higher risk of mortality, and carbapenem resistance exacerbates this process. In a retrospective study from Seoul National University Children's Hospital, carbapenem insensitivity was an important independent risk factor for death from *A. baumannii* bacteremia in PICU patients (11). Early recognition of CRAB infection and mortality determinants is necessary for effective intervention strategies and antibiotic treatment. Although numerous clinical studies regarding CRAB infections have been published, most of them have focused on patients with CRAB bacteremia (11, 12) or ventilator-associated pneumonia (13), and there is a scarcity of studies that investigate CRAB infections in critically ill children. Therefore, this study sought insights into CRAB infection in critically ill children. The aims of this study were (i) to determine the prevalence and potential risk factors for CRAB infection and (ii) to determine the outcomes of CRAB infection and its associated determinants among critically ill children.

## Materials and methods

### Study design and population

This retrospective case-control study was performed at the PICU of the Shanghai Children's Medical Center (SCMC) in China and was approved by the Ethics Committee of Shanghai Children's Medical Center (SCMCIRB-K2023089-1). This hospital is one of the largest pediatric teaching hospitals affiliated with Shanghai Jiaotong University in China, and the PICU is a unit that treats critically ill children with life-threatening conditions such as respiratory failure, heart failure, respiratory cardiac arrest, shock, convulsions, and severe central nervous system depression. All episodes of *A. baumannii* infection that occurred in the PICU were identified through the Hospital Information System (HIS) between January 1st, 2016 and December 31st, 2021. Demographic, clinical, microbiological and outcome data for all episodes were collected. Only the first episode for each patient was included in our cohort, and cases with incomplete medical records were excluded. During the period of this study, patients who developed healthcare-associated infections with *A. baumannii* isolates showing resistance to carbapenems were assigned to a case group while those with isolates sensitive to carbapenems were assigned to a control group.

*A. baumannii* infection was defined as the isolation of *A. baumannii* in at least one culture specimen in patients with concomitant signs and symptoms of infection. All *A. baumannii* infections in this study followed the diagnostic criteria for nosocomial infections (14), combined with specific clinical manifestations and laboratory test results. The outcome was measured as the crude mortality after a positive culture. For statistical purposes, patients who were discharged as terminally ill and refused treatment were considered to have died at the time of hospital discharge.

### Data collection

The demographic and clinical data of enrolled patients were extracted from the hospital information system as follows: age, gender, the circumstances of birth (birth weight, prematurity, vaginal delivery, intrapartum asphyxia, firstborn, breastfeeding), clinical symptoms (fever, icterus, vomiting, diarrhea, thoracodynia, polypnea, cough), comorbidities/underlying disease, clinical outcome (in-hospital mortality, length of hospitalization), coinfection with other bacteria (*S.aureus, E. coli, K. pneumoniae, P. aeruginosa, S.maltophila*) or virus (cytomegalovirus, parainfluenza virus, respiratory syncytial virus, Epstein-Barr virus, influenza virus), medication and intervention therapy within 30 days (parenteral nutrition, central venous/urinary/drainage catheterization, tracheal/gastric intubation, bone marrow aspiration, lumbar puncture glucocorticoid therapy, blood transfusion and antibiotic exposure history), surgery experience within 3 months before infection.

In addition, laboratory tests for C-reactive protein (CRP), white blood cell count (WBC), platelet count (PLT), neutrophil/lymphocyte percentage (%), hemoglobin, albumin, serum urea, serum creatinine, alanine aminotransferase (ALT) and aspartate aminotransferase (AST), were also extracted from the computerized microbiology laboratory databases.

### Microbiology

Bacterial species were firstly identified as *A. baumannii* complex by mass spectrometry (Vitek MS, Biomérieux, France), then were further confirmed by identifying the presence of the *bla*_*OXA-51-like*_ carbapenemase gene that is specific to the *A. baumannii* species. Identification of the causative viral pathogens was by multiplex nucleic acid amplification or the immunological assays for rapid detection of antiviral antibodies or viral antigens.

Antimicrobial susceptibility testing of isolates was performed using a VITEK-2 compact system (bioMérieux, Marcy-l'Étoile, France) with broth microdilution and disk diffusion methods. Results were interpreted in accordance with the 2020 CLSI M100-S30 guidelines (15). The following drugs were tested: amikacin, gentamicin, tobramycin, meropenem, imipenem, ceftazidime, cefepime, cefoperazone/sulbactam, ciprofloxacin, levofloxacin, piperacillin, piperacillin/tazobactam and colistin. For the analysis, *A. baumannii* isolates that were resistant to one or more carbapenems (ertapenem, imipenem, or meropenem) were considered as carbapenem-resistant *A. baumannii*. In addition, *A. baumannii* isolates that were resistant to three or more types of antibacterial drugs simultaneously were identified as multidrug-resistant *A. baumannii* (MDR-AB), and which were resistant to all antibiotics except tigecycline and/or polymyxins were called extensively drug-resistant *A. baumannii* (XDR-AB) (16).

### Statistical analysis

Characteristics of patients are presented with absolute frequencies and percentages for categorical variables, and with median and interquartile range (IQR) for continuous variables. To assess differences, the chi-square test or Fisher's exact test was used to compare the categorical variables; while the Student's *t*-test or Mann–Whitney rank sum test was used to analyze continuous variables. All tests were two-sided, and a *p* < 0.05 was considered as statistically significant. Univariate and multivariate analyses using logistic regression were performed to identify predictors of mortality and risk factors for CRAB infections. All significant variables in univariable comparisons were subsequently selected for inclusion in multivariate analysis. The Bonferroni correction was used to avoid alpha one error for multiple comparisons, and the statistical adjusted significance was accepted at the adjusted *p* < 0.0006. Results were presented as odds ratios (ORs), 95% confidence intervals (CIs), and *p*-values. All statistical analyses were performed with SPSS 26.0 software (IBM Corporation).

## Results

### Prevalence of carbapenem resistance in *A. baumannii* in the PICU from 2016 to 2021

**Figure 1:**
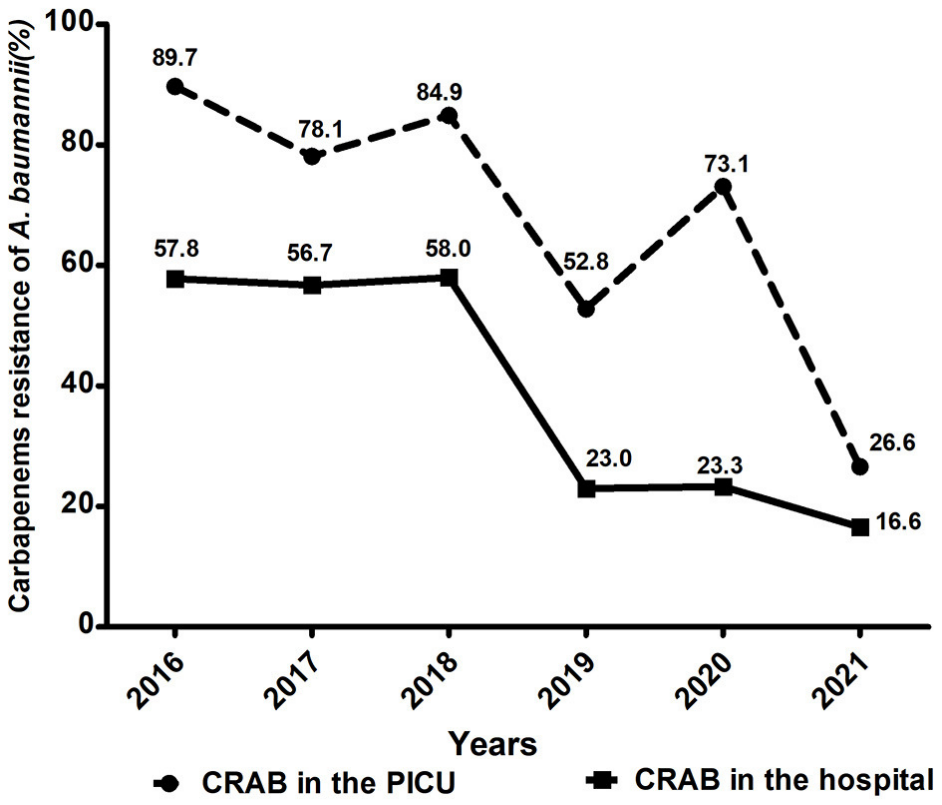
Comparison of trends in carbapenem-resistant *A. baumannii* in the PICU and whole hospital wards from 2016 to 2021.

Through the hospital information system, a total of 372 children admitted to PICU in our hospital were diagnosed with *A. baumannii* infection between January 2016 and December 2021. The overall prevalence of *A. baumannii* infection in the PICU was 9.4% (372/3,974). Among them, the percentage of CRAB reached 78.0% (290/372), which was much higher than that in the whole hospital (54.3%, 660/1,215) over the 6 years. As shown in Figure 1, the proportions of CRAB in the PICU showed decreasing but fluctuating trends, from 89.7% in 2016 to 26.6% in 2021, which is consistent with the trend for CRAB in the hospital.

### Antimicrobial resistance patterns among *A. baumannii* isolates

**Figure 2:**
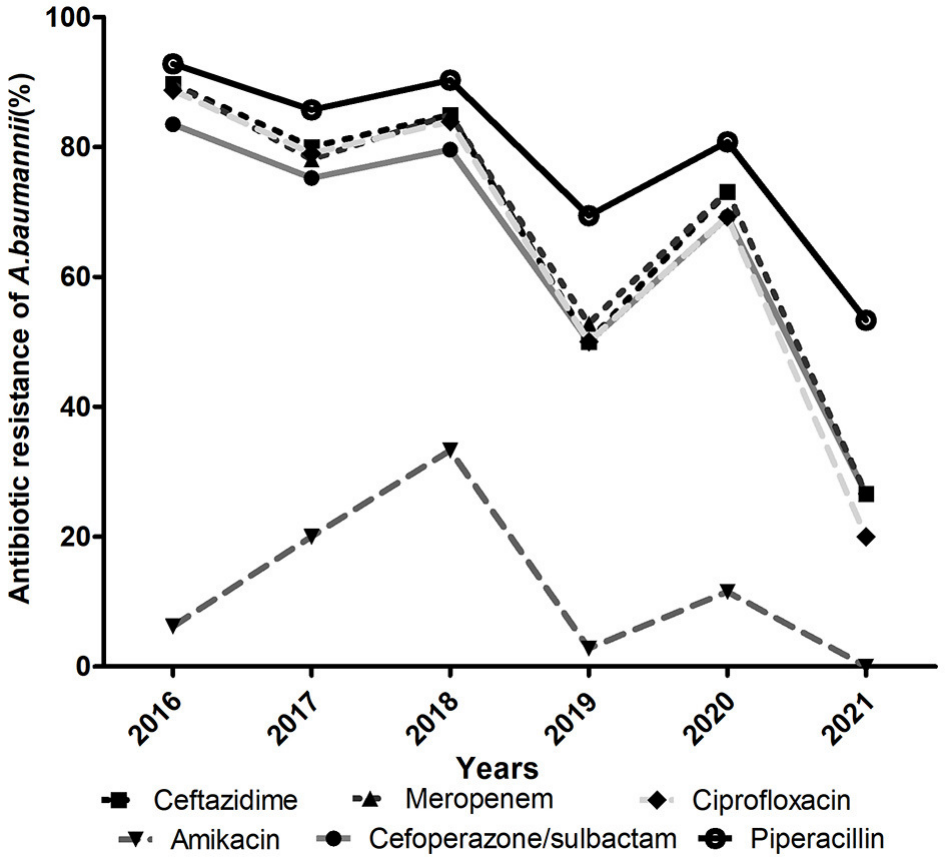
Resistance profile (%) of *A. baumannii* for six main antimicrobials in the PICU from 2016 to 2021.

The antimicrobial resistance profiles of the clinical isolates of *A. baumannii* are summarized in Table 1. Of the 372 isolated strains, 301 (80.9%) were MDR-AB and 26 (7.0%) were XDR-AB. Generally, all *A. baumannii* isolates showed moderate or high rates of resistance to all other antibiotics tested except colistin. Resistance to piperacillin, ceftazidime, ciprofloxacin and cefoperazone-sulbactam displayed decreasing trends from 2016 to 2021 (Figure 2). Specifically, piperacillin (85.5%) was found to be the most resistant antibiotic in this study, followed by cephalosporins (78.2%), carbapenems (78.0%), ciprofloxacin (76.9%), gentamicin (76.3%) and cefoperazone/sulbactam (76.1%). In contrast, amikacin exhibited the highest antimicrobial activity against these strains.

**Table 1:** The Antimicrobial Resistance of *A. baumannii* Strains in this study.

**Antibiotics**	**Total *N* = 372**	**CSAB *N* =82**	**CRAB *N* = 290**	***P*** **-value** ^*^
	**R (** ***n*** **, %)**	**R (** ***n*** **, %)**	**R (** ***n*** **, %)**	
**Aminoglycosides**				
Gentamicin	284 (76.3)	3 (3.7)	281 (96.9)	**<0.001**
Amikacin	62 (16.7)	0 (0.0)	62 (21.4)	**<0.001**
Tobramycin	249 (66.9)	2 (2.4)	247 (85.2)	**<0.001**
**Carbapenems**				
Meropenem	290 (78.0)	0 (0.0)	290 (100.0)	**<0.001**
Imipenem	290 (78.0)	0 (0.0)	290 (100.0)	**<0.001**
**Cephalosporins**				
Ceftazidime	291 (78.2)	4 (4.9)	287 (99.0)	**<0.001**
Cefepime	291 (78.2)	4 (4.9)	287 (99.0)	**<0.001**
**Cephalosporins** + β**-lactamase inhibitors**				
Cefoperazone/sulbactam	274 (73.7)	3 (3.7)	271 (93.4)	**<0.001**
**Fluoroquinolones**				
Ciprofloxacin	286 (76.9)	3 (3.7)	283 (97.6)	**<0.001**
Levofloxacin	170 (45.7)	2 (2.4)	168 (57.9)	**<0.001**
**Penicillins**				
Piperacillin	318 (85.5)	28 (34.1)	290 (100.0)	**<0.001**
**Penicillins** + β**-lactamase inhibitors**				
Piperacillin/tazobactam	283 (76.1)	0 (0.0)	283 (97.6)	**<0.001**
**Lipopeptide**				
Colistin	0 (0.0)	0 (0.0)	0 (0.0)	
MDR-AB	301 (80.9)	11 (13.4)	290 (100.0)	**<0.001**
XDR-AB	26 (7.0)	0 (0.0)	26 (9.0)	**0.005**

CRAB, carbapenem-resistant A. baumannii; CSAB carbapenem-susceptible A. baumannii; MDR, multidrug-resistant; XDR, extensively-drug resistant; R, resistant.

Bold values indicate significant *P* < 0.05.

* Chi square test.

More importantly, antibiotic resistance was even more severe among the CRAB isolates. Except for amikacin, more than 50% of CRAB strains were resistant to other antibiotics tested and a drug resistance rate of >90% was prevalent for piperacillin, ceftazidime, cefepime, ciprofloxacin, gentamicin, piperacillin/tazobactam and cefoperazone/sulbactam. Compared with CRAB, CSAB exhibited a significantly lower resistance rate to all antibiotics and the rate of resistance to piperacillin (34.1%) was the highest.

### Clinical and demographic characteristics of pediatric patients with *A. baumannii* infections

Briefly, the median age of these pediatric patients was 7.2 months, with a male to female ratio of 1.2. Among them, 203(54.6%) were firstborn and 190(51.1%) experienced breastfeeding. Moreover, 179 (48.1%) were born by vaginal delivery and birth weight > 2,500 grams was found in 290 (78.0%).

Polypnea (73.7%), fever (68.5%) and cough (66.4%) were the most common clinical symptoms and respiratory tract infection was the main form of infection. More than 80% of patients suffer from severe underlying diseases or complications, with severe pneumonia being the most common (81.5%). Hypoproteinemia and anemia occurred in 269 (72.3%) and 259 (69.6%) episodes in the progress of the disease, respectively. Additionally, it was found that co-infection of *A. baumannii* and other pathogens (bacteria or viruses) was highly prevalent, occurring in 78.2% of cases.

In the course of treatment, the most frequently used antibiotics were the third-generation cephalosporins (71.2%), followed by carbapenems (55.9%) and glycopeptides (33.5%). Furthermore, 237 (63.7%) cases received hormone therapy, while 299 (80.4%) cases underwent at least one blood transfusion within 30 days before infection. In addition, 338(90.9%) children were exposed to some sort of invasive procedures in a month prior to *A. baumannii* isolation, including 79.8% gastric intubation, 79.3% endotracheal intubation, 54.0% indwelling urinary catheters, 24.7% central venous catheters, and 9.7% drainage tubes. Bone marrow and lumbar punctures were performed in 10.5% and 16.1% of patients, respectively. Nearly half (44.4%) of the patients had undergone surgery within 3 months prior to infection.

### Risk factors for the development of CRAB infections in the PICU

In order to determine the risk factors for CRAB infection in critically ill children, a retrospective case-control analysis was conducted, including 290 children with CRAB infection and 82 children with CSAB infection.

The demographic and clinical characteristics of children with CRAB and CSAB infections are summarized in Table 2. On univariate analysis, children with CRAB infection tend to show more symptoms of fever (72.1 vs. 56.1%, *p* = 0.006) and polypnea (77.2 vs. 61.0%, *p* = 0.003), had a higher proportion of hemoglobin <110 g/L (91.7 vs. 70.7%, *p* < 0.0006), neutrophile>70% (37.6 vs. 22.0%, *p* = 0.008) and have lower proportion of vomiting (21.0 vs. 34.1, *p* = 0.014), neutrophile <50% (31.0 vs. 50.0%, *p* = 0.002), ALT <13 U/L(1.7 vs. 8.5%, *p* = 0.002) than those with CSAB infection. However, after the Bonferroni correction, only hemoglobin < 110 g/L remained significantly associated with CRAB infection (*p* < 0.0006).

**Table 2:** Baseline and clinical characteristics of 372 hospitalized children with *A.baumannii* infections.

**Characteristics**	**Total *N* = 372**	**CSAB *N* = 82**	**CRAB *N* = 290**	*P*-value^*^
**Demographics**				
Male gender	206 (55.4)	47 (57.3)	159 (54.8)	0.689
Age (years)	0.6 (0.4–2.5)	1.0 (0.3–6.5)	0.6 (0.4–2.1)	0.053^**^
Birth weight (Kg)	3.1 (2.5–3.5)	3.2 (2.6–3.5)	3.0 (2.5–3.5)	0.065^**^
Prematurity	43 (11.6)	6 (7.3)	37 (12.8)	0.174
Vaginal delivery	179 (48.1)	46 (56.1)	133 (45.9)	0.101
Breastfeeding	190 (51.1)	47 (57.3)	143 (49.3)	0.200
Firstborn	203 (54.6)	45 (54.9)	158 (54.4)	0.949
Intrapartum asphyxia	21 (5.6)	3 (3.7)	18 (6.2)	0.587^***^
**Specimen type**				
Sputum	263 (70.7)	65 (79.3)	198 (68.3)	0.053
Blood	31 (8.3)	6 (7.3)	25 (8.6)	0.706
Urine	3 (0.8)	0 (0)	3 (1.0)	1.000^***^
Others	75 (20.2)	11 (13.4)	64 (22.1)	0.085
**Clinical symptoms**				
Polypnea	274 (73.7)	50 (61.0)	224 (77.2)	**0.003**
Fever	255 (68.5)	46 (56.1)	209 (72.1)	**0.006**
Vomiting	89 (23.9)	28 (34.1)	61 (21.0)	**0.014**
Icterus	139 (37.4)	26 (31.7)	113 (39.0)	0.230
Diarrhea	62 (16.7)	16 (19.5)	46 (15.9)	0.434
Thoracodynia	7 (1.9)	1 (1.2)	6 (2.1)	1.000^***^
Cough	247 (66.4)	53 (64.6)	194 (66.9)	0.701
**Laboratory index**				
C-reactive protein> 8 mg/L	210 (56.5)	42 (51.2)	168 (57.9)	0.279
Hemoglobin < 110 g/L	324 (87.1)	58 (70.7)	266 (91.7)	**<0.0006**
Neutrophile > 70%	127 (34.1)	18 (22.0)	109 (37.6)	**0.008**
Neutrophile < 50 %	131 (35.2)	41 (50.0)	90 (31.0)	**0.002**
Lymphocyte > 40 %	106 (28.5)	30 (36.6)	76 (26.2)	0.066
Lymphocyte < 20 %	131 (35.2)	22 (26.8)	109 (37.6)	0.072
Platelet < 100 × 10∧9/L	90 (24.2)	21 (25.6)	69 (23.8)	0.734
WBC > 15.0 × 10∧9/L	61 (16.4)	14 (17.1)	47 (16.2)	0.852
Albumin < 35 g/L	207 (55.6)	42 (51.2)	165 (56.9)	0.361
ALT > 69 U/L	83 (22.3)	19 (23.2)	64 (22.1)	0.832
ALT < 13 U/L	12 (3.2)	7 (8.5)	5 (1.7)	**0.006** ^***^
AST < 15 U/L	6 (1.6)	0 (0)	6 (2.1)	0.346^***^
AST > 46 U/L	186 (50.0)	41 (50.0)	145 (50.0)	1.000
Urea < 3.2 mmol/L	105 (28.2)	26 (31.7)	79 (27.2)	0.428
Urea > 7.1 mmol/L	79 (21.2)	19 (23.2)	60 (20.7)	0.628
Creatinine < 9 umol/L	18 (4.8)	4 (4.9)	14 (4.8)	1.000^***^
Creatinine > 88 umol/L	16 (4.3)	6 (7.3)	10 (3.4)	0.132^***^
**Pathogenies co-infections**				
*K. pneumoniae*	36 (9.6)	4 (4.9)	32 (11.0)	0.096
*S. aureus*	21 (5.6)	5 (6.1)	16 (5.5)	0.790^***^
*P. aeruginosa*	24 (6.5)	3 (3.7)	21 (7.2)	0.244
*S.maltophila*	38 (10.2)	11 (13.4)	27 (9.3)	0.279
*E. coli*	9 (2.4)	1 (1.2)	8 (2.8)	0.690^***^
Cytomegalovirus	20 (5.4)	2 (2.4)	18 (6.2)	0.268^***^
Parainfluenza virus	12 (3.2)	3 (3.7)	9 (3.1)	0.731^***^
Respiratory syncytial virus	13 (3.5)	4 (4.9)	9 (3.1)	0.495^***^
Epstein-Barr virus	8 (2.2)	3 (3.7)	5 (1.7)	0.382^***^
Influenza virus	5 (1.3)	1 (1.2)	4 (1.4)	1.000^***^
**Complications/underlying disease**				
Severe Pneumonia	303 (81.5)	48 (58.5)	255 (87.9)	**<0.0006**
Hypoproteinemia	269 (72.3)	44 (53.7)	225 (77.6)	**<0.0006**
Anemia	259 (69.6)	43 (52.4)	216 (74.5)	**<0.0006**
Respiratory failure	225 (60.5)	51 (62.2)	174 (60.0)	0.720
Septic shock	52 (14.0)	11 (13.4)	41 (14.1)	0.868
Leukemia	29 (7.8)	9 (11.0)	20 (6.9)	0.224
Congenital heart disease	144 (38.7)	27 (32.9)	117 (40.3)	0.223
**Treatments**				
Invasive operation before infection (within 30 days)	338 (90.9)	52 (63.4)	286 (98.6)	**<0.0006**
Gastric intubation	297 (79.8)	40 (48.8)	257 (88.6)	**<0.0006**
Tracheal intubation	295 (79.3)	41 (50.0)	254 (87.6)	**<0.0006**
Urinary catheterization	201 (54.0)	26 (31.7)	175 (60.3)	**<0.0006**
Drainage intubation	36 (9.7)	3 (3.7)	33 (11.4)	**0.037**
Central venous catheterization	92 (24.7)	16 (19.5)	76 (26.2)	0.215
Bone marrow aspiration	39 (10.5)	11 (13.4)	28 (9.7)	0.327
Lumbar puncture	60 (16.1)	13 (15.9)	47 (16.2)	0.939
Previous surgery (within 3 months)	165 (44.4)	27 (32.9)	138 (47.6)	**0.018**
Parenteral nutrition	96 (25.8)	12 (14.6)	84 (29.0)	**0.009**
Blood transfusion (within 30 days)	299 (80.4)	46 (56.1)	253 (87.2)	**<0.0006**
Corticosteroid therapy (within 30 days)	237 (63.7)	45 (54.9)	192 (66.2)	0.060
**Antibiotic use before infection (within 30 days)**				
3^rd^ Cephalosporins	265 (71.2)	50 (61.0)	215 (74.1)	**0.020**
Carbapenems	208 (55.9)	23 (28.0)	185 (63.8)	**<0.0006**
Glycopeptides	163 (43.8)	19 (23.2)	144 (49.7)	**<0.0006**
Macrolides	88 (23.7)	12 (14.6)	76 (26.2)	**0.029**
Fluoroquinolones	30 (8.1)	2 (2.4)	28 (9.7)	**0.034**
Aminoglycosides	43 (11.6)	10 (12.2)	33 (11.4)	0.838
Penicillins	47 (12.6)	7 (8.5)	40 (13.8)	0.206
Tetracyclines	14 (3.8)	3 (3.7)	11 (3.8)	1.000^***^
Antifungal agents	113 (30.4)	13 (15.9)	100 (34.5)	**0.001**
Length of hospitalization (days)	29 (19–43)	25 (12–37)	31 (20–44)	**0.002** ^**^
Hospitalization expenses	114,161 (62,262–175,644)	93,393 (30,166–161,268)	116,316 (70,941–178,666)	**0.013** ^**^
**Outcome**				
Hospital mortality	115 (30.9)	24 (29.3)	91 (31.4)	0.715

CRAB, carbapenem-resistant A.baumannii; CSAB, carbapenem-susceptible A.baumannii; ALT, Alanine transaminase; WBC, white blood cell.

Bold values indicate significant p < 0.05.

* Chi square test;

** Mann–Whitney rank sum test;

*** Fisher's exact test. The statistical adjusted significance was accepted at the adjusted p < 0.0006 after Bonferroni correction.

As shown in Table 2, higher rates of severe pneumonia (87.9 vs. 58.5%, *p* < 0.0006), anemia (74.5 vs. 52.4%, *p* < 0.0006) and hypoproteinemia (77.6 vs. 53.7%, *p* < 0.0006) were observed in patients with CRAB infection. Furthermore, compared with CSAB-infected patients, more patients with CRAB infection experienced invasive procedures including tracheal intubation (87.6 vs. 50.0%, *p* < 0.0006), urinary catheterization (60.3 vs. 31.7%, *p* < 0.0006) and gastric intubation (87.6 vs. 50.0, *p* < 0.0006). In addition, more patients suffered from CRAB infection received a blood transfusion (87.2 vs. 56.1%, *p* < 0.0006) and specific antibiotic treatments before *A. baumannii* isolation, including carbapenems (63.8 vs. 28.0%, *p* < 0.0006), and glycopeptides (49.7 vs. 23.2%, *p* < 0.0006).

To further evaluate the independent predictors of CRAB acquisition in pediatric patients, a multivariate logistic regression analysis was performed (Table 3). Of note, receiving invasive operation (OR = 9.412, *p* = 0.001), was found to be the best independent risk factor for the development of CRAB infection. Hemoglobin < 110g/L (OR = 3.049, *p* = 0.005), severe pneumonia (OR = 3.235, *p* = 0.001), previous exposure to carbapenems (OR = 2.543, *p* = 0.003) and presence of gastric intubation within 1 month before infection (OR = 2.478, *p* = 0.026) were also significantly associated with CRAB acquisition.

**Table 3:** Risk factors for carbapenem-resistant *A. baumannii* infection with multivariate logistic regression analysis.

	***p*-value**	**OR**	**95% CI**
Invasive operation (within 30 days)	**0.001**	9.412	2.547–34.785
Gastric intubation	**0.026**	2.478	1.117–5.499
Prior carbapenems exposure	**0.003**	2.543	1.367–4.729
Severe pneumonia	**0.001**	3.235	1.627–6.431
Hemoglobin < 110 g/L	**0.005**	3.049	1.410–6.593

CRAB, carbapenem-resistant Acinetobacter baumannii; CSAB carbapenem-susceptible Acinetobacter baumannii. OR, odds ratio; CI, confidence interval.

Bold values indicate significant p < 0.05.

### Risk factors affecting the prognosis of critically ill children with *A. baumannii* infection

Of the 372 patients with *A. baumannii* infection, 115 patients (30.9%) died in the hospital. Univariate analyses of risk factors associated with mortality are shown in Table 4. Children who had a poor outcome were older in median age (*p* < 0.0006), had a higher proportion of icterus (*p* < 0.0006), CRP>8 mg/L (*p* < 0.0006), lymphocyte < 20% (*p* < 0.0006), PLT < 100 x 10^9^/L (*p* < 0.0006) and AST>46U/L (*p* < 0.0006). Moreover, higher rates of septic shock (*p* < 0.0006) and bone marrow puncture (*p* < 0.0006) were more frequent in non-survivors. Besides, previous exposure to glycopeptides (*p* = 0.016), aminoglycosides (*p* = 0.019) and antifungal drugs (*p* = 0.001) in non-survivors was higher than those in survivors. However, there was no significant association between previous antibiotic exposure and clinical outcome after the Bonferroni correction.

**Table 4:** Univariate analysis for factors associated with mortality of children with *A. baumannii* infection.

**Characteristics**	**Total *N* = 372**	**Died *N* = 115**	**Survivor *N* = 257**	*P*-value^*^
**Demographics**				
Male gender	206 (55.4)	66 (57.4)	140 (54.5)	0.601
Age (years)	0.6 (0.4–2.5)	1.3 (0.4–5.0)	0.6 (0.3–1.7)	<0.0006**
Birth weight (Kg)	3.1 (2.5–3.5)	3.2 (2.7–3.6)	3.0 (2.5–3.5)	0.132^**^
Prematurity	43 (11.6)	14 (12.2)	29 (11.3)	0.804
Vaginal delivery	179 (48.1)	52 (45.2)	127 (49.4)	0.454
Breastfeeding	190 (51.1)	59 (51.3)	131 (51.0)	0.953
Firstborn	203 (54.6)	65 (56.5)	138 (53.7)	0.613
Intrapartum asphyxia	21 (5.6)	5 (4.3)	16 (6.2)	0.468
**Specimen type**				
Sputum	263 (70.7)	85 (73.9)	178 (69.3)	0.362
Blood	31 (8.3)	17 (14.8)	14 (5.4)	**0.003**
Urine	3 (0.8)	1 (0.9)	2 (0.8)	1.000^***^
Others	79 (21.2)	21 (18.3)	58 (22.6)	0.348
**Clinical symptoms**				
Fever	255 (68.5)	89 (77.4)	166 (64.6)	**0.014**
Icterus	139 (37.4)	58 (50.4)	81 (31.5)	**<0.0006**
Vomiting	89 (23.9)	37 (32.2)	52 (20.2)	**0.013**
Diarrhea	62 (16.7)	23 (20.0)	39 (15.2)	0.249
Thoracodynia	7 (1.9)	2 (1.7)	5 (1.9)	1.000^***^
Polypnea	274 (73.7)	85 (73.9)	189 (73.5)	0.940
Cough	247 (66.4)	74 (64.3)	173 (67.3)	0.576
**Laboratory index**				
Hemoglobin < 110 g/L	324 (87.1)	100 (87.0)	224 (87.2)	0.957
Hemoglobin < 90 g/L	200 (53.8)	69 (60.0)	131 (51.0)	0.107
C-reactive protein> 8 mg/L	210 (56.5)	83 (72.2)	127 (49.4)	**<0.0006**
Albumin < 35 g/L	207 (55.6)	77 (67.0)	130 (50.6)	**0.003**
Neutrophile > 70%	127 (34.1)	51 (44.3)	76 (29.6)	**0.005**
Neutrophile < 50 %	131 (35.2)	35 (30.4)	96 (37.4)	0.197
Lymphocyte > 40 %	106 (28.5)	26 (22.6)	80 (31.1)	0.092
Lymphocyte < 20 %	131 (35.2)	57 (49.6)	74 (28.8)	**<0.0006**
Platelet < 100 × 10∧9/L	90 (24.2)	46 (40.0)	44 (17.1)	**<0.0006**
WBC > 15.0 × 10∧9/L	61 (16.4)	23 (20.0)	38 (14.8)	0.209
ALT > 69 U/L	83 (22.3)	33 (28.7)	50 (19.5)	**0.048**
ALT < 13 U/L	12 (3.2)	0 (0)	12 (4.7)	0.021^***^
AST < 15 U/L	6 (1.6)	2 (1.7)	4 (1.6)	1.000^***^
AST > 46 U/L	186 (50.0)	74 (64.3)	112 (43.6)	**<0.0006**
Urea < 3.2 mmol/L	105 (28.2)	22 (19.1)	83 (32.3)	**0.009**
Urea > 7.1 mmol/L	79 (21.2)	36 (31.3)	43 (16.7)	**0.001**
Creatinine < 9 umol/L	18 (4.8)	5 (4.3)	13 (5.1)	0.768
Creatinine > 88 umol/L	16 (4.3)	11 (9.6)	5 (1.9)	0.002^***^
**Antimicrobial resistance**				
CRAB	290 (78.0)	91 (79.1)	199 (77.4)	0.715
MDR-AB	301 (80.9)	95 (82.6)	206 (71.8)	0.578
XDR-AB	26 (7.0)	6 (5.2)	20 (7.8)	0.370
**Pathogenies co-infections**				
*K. pneumoniae*	36 (9.7)	9 (7.8)	27 (10.5)	0.419
*S. aureus*	21 (5.6)	7 (6.1)	14 (5.4)	0.805
*P. aeruginosa*	24 (6.5)	7 (6.1)	17 (6.6)	0.848
*S.maltophila*	38 (10.2)	9 (7.8)	29 (11.3)	0.309
*E. coli*	9 (2.4)	2 (1.7)	7 (2.7)	0.727^***^
Cytomegalovirus	20 (5.4)	4 (3.5)	16 (6.2)	0.278
Parainfluenza virus	12 (3.2)	3 (2.6)	9 (3.5)	0.761^***^
Respiratory syncytial virus	13 (3.5)	3 (2.6)	10 (3.9)	0.762^***^
Epstein-Barr virus	8 (2.2)	5 (4.3)	3 (1.2)	0.113^***^
Influenza virus	5 (1.3)	2 (1.7)	3 (1.2)	0.647^***^
**Complications/underlying disease**				
Septic shock	52 (14.0)	31 (27.0)	21 (8.2)	**<0.0006**
Leukemia	29 (7.8)	17 (14.8)	12 (4.7)	**0.001**
Hypoproteinemia	269 (72.3)	92 (80.0)	177 (68.9)	**0.027**
Anemia	261 (70.2)	90 (78.3)	171 (66.5)	**0.022**
Severe Pneumonia	303 (81.5)	96 (83.5)	207 (80.5)	0.501
Respiratory failure	225 (60.5)	71 (61.7)	154 (59.9)	0.740
Congenital heart disease	144 (38.7)	37 (32.2)	107 (41.6)	0.083
**Treatments**				
Invasive operation (within 30 days)	338 (90.9)	103 (89.6)	235 (91.4)	0.562
Bone marrow aspiration	39 (10.5)	22 (19.1)	17 (6.6)	**<0.0006**
Central venous catheterization	92 (24.7)	40 (34.8)	52 (20.2)	**0.003**
Tracheal intubation	295 (79.3)	89 (77.4)	206 (80.2)	0.543
Urinary catheterization	201 (54.0)	66 (57.4)	135 (52.5)	0.385
Drainage intubation	36 (9.7)	13 (11.3)	23 (8.9)	0.478
Lumbar puncture	60 (16.1)	19 (16.5)	41 (16.0)	0.890
Gastric intubation	297 (79.8)	88 (76.5)	209 (81.3)	0.286
Previous surgery (within 3 months)	165(44.4)	42 (36.5)	123 (47.9)	**0.042**
Parenteral nutrition	96 (25.8)	38 (33.0)	58 (22.6)	**0.033**
Blood transfusion (within 30 days)	299 (80.4)	96 (83.5)	203 (79.0)	0.314
Corticosteroid therapy (within 30 days)	237 (63.7)	73 (63.5)	164 (63.8)	0.950
**Previous antibiotic therapy (within 30 days)**				
3rd Cephalosporins	265 (71.2)	79 (68.7)	186 (72.4)	0.469
Carbapenems	208 (55.9)	72 (62.6)	136 (52.9)	0.082
Penicillins	47 (12.6)	11 (9.6)	36 (14.0)	0.233
Macrolides	88 (23.7)	24 (20.9)	64 (24.9)	0.398
Tetracyclines	14 (3.8)	7 (6.1)	7 (2.7)	0.141^***^
Fluoroquinolones	30 (8.1)	10 (8.7)	20 (7.8)	0.765
Glycopeptides	163 (43.8)	61 (53.0)	102 (39.7)	**0.016**
Aminoglycosides	43 (11.6)	20 (17.4)	23 (8.9)	**0.019**
Antifungal agents	113 (30.4)	49 (42.6)	64 (24.9)	**0.001**
Length of hospitalization(days)	29 (19–43)	23 (13–41)	32 (21–46)	0.001^**^
Hospitalization expenses	114,161 (62,262–175,644)	114,626 (53,733–216,367)	113,931 (66,924–166,565)	0.624^**^

CRAB, carbapenem-resistant A.baumannii; CSAB, carbapenem-susceptible A.baumannii; ALT, Alanine transaminase; WBC, white blood cell.

Bold values indicate significant p < 0.05.

* Chi square test;

** Mann–Whitney rank sum test;

*** Fisher's exact test.

The statistical adjusted significance was accepted at the adjusted p < 0.0006 after Bonferroni correction.

In the final multivariable model, septic shock (OR = 2.992, *p* = 0.001), AST>46U/L (OR = 2.015, *p* = 0.005), bone marrow aspiration (OR = 2.704, *p* = 0.008), lymphocyte <20 % (OR = 1.992, *p* = 0.006) and age (OR = 1.094, *p* = 0.002) at infection onset were independent risk factors for mortality due to *A. baumanni* infection in the PICU (Table 5). However, mortality was not higher in patients with CRAB infection than in those with CSAB infection.

**Table 5:** Risk factors for mortality of *Acinetobacter baumannii* infection with multivariate logistic regression analysis.

	***p*-value**	**OR**	**95% CI**
Septic shock	**0.001**	2.992	1.508–5.661
AST >46 U/L	**0.005**	2.015	1.241–3.271
Lymphocyte < 20 %	**0.006**	1.992	1.219–3.255
Age	**0.002**	1.094	1.035–1.157
Bone marrow aspiration	**0.008**	2.704	1.291–5.665

CRAB, carbapenem-resistant A. baumannii; CSAB, carbapenem-susceptible A. baumannii; ALT, Alanine transaminase. OR, odds ratio; CI, confidence interval.

Bold values indicate significant p < 0.05.

## Discussion

CRAB infections are becoming one of the most cause of public healthcare threats worldwide (17) and carry an increased risk of infection-associated death for children. In our study, the rate of CRAB among *A. baumannii* infection in the PICU has reached as high as 78.0%, which is higher than in most countries in Europe (18, 19) and North America (20). A study of surveillance data from European Union countries found that the population-weighted mean proportion of carbapenem-non-susceptible *Acinetobacter spp*. isolates in ICUs reached 54.0% [95% CI 47.6–60.3%] (19). Data from 70 US medical centers in 2018–2020 showed the proportions of carbapenem-non-susceptible *A. baumannii* in ICUs and non-ICUs were 29.9% and 27.8%, respectively (20). Consequently, *A. baumannii* infection in the ICU should draw more attention in consideration of high carbapenem resistance rate. In addition to carbapenems resistance, *A. baumannii* showed high rates of resistance to cephalosporins, β-Lactam inhibitors, penicillins, quinolones, aminoglycosides and 81.0% exhibited multidrug resistance phenotypes. Moreover, CRAB showed significantly higher rates of resistance to all tested antimicrobials than CSAB, with the exception of colistin. High-resistance rates in CRAB will inevitably complicate the treatment of severe infections in critically ill patients and should accelerate efforts toward effective antimicrobial stewardship and infection control strategies.

We also observed a significant reduction in the proportion of CRAB from 2016 to 2021. The decrease in the prevalence of CRAB after 2018 may be attributable: (1) to strengthen environmental monitoring and disinfection of wards and offices, as well as staff disinfection during the COVID-19 pandemic; (2) Both medical staff and patients strictly complied with medical guidance and took good personal protection to reduce the risk of bacterial transmission during this time period; (3) Due to quarantine restrictions, fewer patients were admitted to the PICU and are less likely to develop nosocomial CRAB infections.

The present study identified several independent risk factors for developing CRAB infection in critically ill children, including invasive operation, gastric intubation, prior carbapenems exposure, severe pneumonia and low hemoglobin level. Increasing evidence indicates that prior invasive procedures, including the presence of mechanical ventilation, endotracheal intubation, central venous or umbilical artery catheterization, and abdominal drainage catheterization are associated with a higher risk of CRAB infections (13, 22–24). Invasive procedures can destroy intact skin, which may create opportunities for CRAB strains to colonize the skin or even enter internal tissues. Furthermore, CRAB strains can exhibit the natural ability to form biofilms on the catheter, causing infection by migrating into the epithelial cells of the internal tissue (21). Consistent with many other studies, we identified a series of invasive procedures, including tracheal intubation (*p* < 0.001), urinary catheterization (*p* < 0.001), gastric intubation (*p* < 0.001), drainage intubation (*p* = 0.037) and surgery (*p* = 0.018) were associated with CRAB infection in univariable analysis. However, only the use of gastric intubation remained as an independent risk factor. Clinically, most patients with gastric intubation have poor nutrition and gastrointestinal dysfunction, which increases the risk of intestinal bacterial translocation, including CRAB. Therefore, our results emphasize the importance of the strict application of catheterization and nursing in children.

Antibiotic exposure remains one of the most important risk factors for the acquisition of CRAB. A study from Thailand found that prior use of cephalosporins increased the risk of CRAB ventilator-associated pneumonia in neonates (13). Previous exposure to aminoglycosides or carbapenems has long been considered as a high-risk factor contributing to CRAB infection in hospitalized patients (25–27). As identified in previous studies, prior carbapenems exposure is a critical risk factor for developing CRAB infections in this study. In fact, the increased number of infections due to multidrug-resistant *Acinetobacter* in the PICU requires empirical broad-spectrum antimicrobials, especially carbapenems. Long-term and high frequency use of such antibiotics makes *A. baumannii* undergo gene mutation or acquire new resistance genes to survive under the pressure of antibiotics, resulting in changes in the resistance characteristics of bacteria (28). This phenomenon was mainly observed when the treatment had occurred between 6 months (ceftazidime) and 9 months (imipenem and levofloxacin) prior (29). Considering these factors, hospitals should strengthen antibiotic management and pay more attention to patients with CSAB infection who have prior exposure to carbapenems.

Risk factors for nosocomial acquisition of antibiotic-resistant microorganisms include not only prior exposure to antibiotics, but also variables related to host defense and infection control measures. Previous reports have shown the disease severity [Acute Physiology and Chronic Health Evaluation (APACHE) II score] was a significant risk factor for the acquisition of CRAB (30, 31). Compatible with these reports, our study revealed that patients who acquired CRAB infection often had severe pneumonia and low hemoglobin level. They had a weaker immune system and were more vulnerable to invasive maneuvers and multi-drug resistant bacteria. The ICU environment with high CRAB colonization and a crowd of ventilated patients may serve as a reservoir for cross-transmission and provoke exogenous acquisition of CRAB.

To explore the possible influence of CRAB infection on the outcome of critically ill children, their demographic and clinical characteristics were systematically evaluated. In our study, the crude in-hospital mortality of the 372 patients with *A. baumannii* infection was 30.9% (115/372), which was significantly higher than the 30-day all-cause mortality rate (22.4%) of patients with *A. baumannii* complex bacteraemia reported in the Chinese Antimicrobial Resistance Surveillance of Nosocomial Infections (CARES) Network (22). This is partly because the patient has serious underlying diseases or complications and/or inappropriate treatment.

Predictors of mortality have been extensively studied in patients with *A. baumannii* bacteremia. A multicenter retrospective study in Taiwan has revealed that carbapenem resistance conferred enhanced risks for mortality in patients with *A. baumannii* complex bacteremia (32). According to another national prospective study from CARES, carbapenem resistance was also reported as an independent variable for 30-day all-cause mortality in patients with *A. baumannii* bacteremia (23). Interestingly, a retrospective study in the United States found the mortality was significantly higher in patients with non-CSAB bacteremia than in those with CSAB bacteremia, but they no longer found a significant association between CRAB and increased risk of death after adjusting for potential confounders (33). In our study, we did not observe that CRAB was associated with an apparent unfavorable impact on morbidity and mortality in *A. baumannii* infection. Currently, there is ongoing controversy as to whether carbapenem resistance results in an increased risk of mortality in patients infected with *A. baumannii* (34). These discrepancies may be attributed in part to differences in the study population or the sample size, or an overall lack of statistical power.

In addition to carbapenem resistance, disease severity (such as high APACHE II scores and shock state) has also been reported in many studies to be independently associated with poor outcomes in patients with *A. baumannii* bacteremia (35, 36). Consistent with these studies, we found septic shock, AST > 46 U/L, lymphocyte < 20 %, and age were independently associated with poor prognosis in *A. baumannii* infection. Septic shock is a life-threatening condition that results from the combined effects of bacteria and the host's chemical mediators of inflammation (37). It is well known that mortality increases with each hour that appropriate antimicrobial therapy is delayed in patients with septic shock (38). Serum levels of aspartate aminotransferases (AST) has been regarded as an important marker of liver injury. Its serum concentrations are highly variable and are affected by a myriad of factors, including liver function, dietary protein intake, metabolic rate, medication status, and hydration status (39), etc. These factors often occur in ICU patients, leading to their immune deficiency, which supports that the patient's physical condition is closely related to their survival. Lymphocytes are the primary cells of the immune system including T cells, B cells and natural killer cells. These cells are responsible for producing cytotoxic T cells or antibodies that directly eliminating invading pathogens (viruses, bacteria), and regulate the immune response (40). Low concentrations of lymphocytes suppressed natural and adaptive immunity, further played an adverse role in the disease progression. An International Study from China and Germany indicated lymphocytes (%) was one of five clinical predictors of the mortality in patients with COVID-19 infection (41). Previous studies have reported age (>65 years) as an independent risk factor for *A. baumannii* infection-related deaths in hospitalized patients and deaths increased by a factor of 1.04 with every additional year of age (42). Similar to this result, our founding suggests that age contributing to *A. baumannii* infection-related deaths in critically ill children. This may be due to their low immunity and long-term underlying diseases without effective treatment and control.

Bone marrow aspiration is a valuable procedure commonly utilized for evaluation of hematologic abnormalities and suspected infection in patients with fever of unknown origin (43). This invasive procedure also destroy intact skin and probably create conditions for *A. baumannii* to colonize the skin and even enter the bone marrow, leading to severe bone marrow infections that threaten the patient's life. A 51-year-old man with chronic lymphocytic leukemia developed acute osteomyelitis and bloodstream infection caused by *Staphylococcus aureus* after bone marrow aspiration and renal aspiration biopsy (44). Therefore, the strict application and care of bone marrow aspiration in children are closely related to the prognosis of patients.

This study focused on the clinical characteristics of *A. baumannii* infection in critically ill children in China. The main limitations were its single-center retrospective nature and the relatively small sample size, which may affect the power of the analysis to identify significant factors. Furthermore, we could not perform the matching process, which would be useful in limiting selection bias. This was mainly due to the relatively small number of patients in the control group and the fact that both groups of patients had similar underlying diseases. Finally, there is no data exploring the genotype and/or resistance mechanism of CRAB isolates, which would shed new light on the spread of CRAB.

In conclusion, this study highlights high rates of CRAB and MDRAB in critically ill children with *A. baumannii* infection. Children with invasive operation, gastric intubation, prior carbapenems exposure, severe pneumonia and low hemoglobin level were more likely to develop CRAB infections. Further, the severity of illness and bone marrow aspiration were independently associated with the mortality due to *A. baumanni* infection among critically ill children. Based on these above risk factors, the combined application of targeted interventions and rational antibiotic therapy could effectively control the emergence and spread of CRAB in the ICU. Of course, these control measures are crucial and must be extended to other healthcare settings for the eradication of CRAB, which will ultimately improve the outcomes for *A. baumannii* infection in the world.

## Data availability statement

The original contributions presented in the study are included in the article/Supplementary material, further inquiries can be directed to the corresponding authors.

## Ethics statement

The studies involving humans were approved by the Ethics Committee of Shanghai Children's Medical Center. The studies were conducted in accordance with the local legislation and institutional requirements. The Ethics Committee/Institutional Review Board waived the requirement of written informed consent for participation from the participants or the participants' legal guardians/next of kin because this was a retrospective observational study, and it did not cause any disruption to patients.

## Author contributions

YZ: Data curation, Formal analysis, Investigation, Software, Writing—review & editing. GX: Conceptualization, Formal analysis, Investigation, Methodology, Writing—review & editing. FM: Conceptualization, Formal analysis, Investigation, Methodology, Writing—review & editing. WH: Data curation, Investigation, Writing—review & editing. HW: Methodology, Project administration, Supervision, Writing—review & editing. XW: Project administration, Supervision, Writing—original draft, Writing—review & editing, Methodology, Funding acquisition.
