# Supplementary material for: Insights into the epidemiology, risk factors, and clinical outcomes of carbapenem-resistant Acinetobacter baumannii infections in critically ill children
Source: Front Public Health. 2023 Nov 30;11:1282413. doi: 10.3389/fpubh.2023.1282413 (PMC10720883; doi:10.3389/fpubh.2023.1282413)
Supplement: Supplementary file 1 [file Table_1.doc]

Supplemental table 1 Clinical and demographic characteristics of pediatric patients with *A. baumannii* infections.

| **Characteristics** | **Total N=372** |
| --- | --- |
| **Demographics** |  |
| Male gender | 206 (55.4) |
| Age(years) | 0.6 (0.4-2.5) |
| **Birth weight(g)** | 3.1 (2.5-3.5) |
| **Prematurity** | 43 (11.6) |
| **Vaginal delivery** | 179 (48.1) |
| **Breastfeeding** | 190 (51.1) |
| **Firstborn** | 203 (54.6) |
| **Intrapartum asphyxia** | 21 (5.6) |
| **Specimen type** |  |
| Sputum | 263 (70.7) |
| Blood | 31 (8.3) |
| Urine | 3 (0.8) |
| Others | 75 (20.2) |
| **Clinical symptoms** |  |
| Polypnea | 274 (73.7) |
| Fever | 255 (68.5) |
| Cough | 247 (66.4) |
| Vomiting | 89 (23.9) |
| Icterus | 139 (37.4) |
| Diarrhea | 62 (16.7) |
| Thoracodynia | 7 (1.9) |
| **Pathogenies co-infections** |  |
| *K. pneumoniae* | 36 (9.6) |
| *S. aureus* | 21 (5.6) |
| *P. aeruginosa* | 24 (6.5) |
| *S.maltophila* | 38 (10.2) |
| *[E. coli](../../Documents/WeChat%20Files/plot/AppData/Local/youdao/dict/Application/9.0.1.1/resultui/html/index.html#javascript:;)* | 9 (2.4) |
| Cytomegalovirus | 20 (5.4) |
| Parainfluenza virus | 12 (3.2) |
| Respiratory syncytial virus | 13 (3.5) |
| Epstein-Barr virus | 8 (2.2) |
| Influenza virus | 5 (1.3) |
| **Complications / Underlying disease** |  |
| Severe Pneumonia | 303 (81.5) |
| Hypoproteinemia | 269 (72.3) |
| Anemia | 259 (69.6) |
| Respiratory failure | 225 (60.5) |
| Congenital heart disease | 144 (38.7) |
| Septic shock | 52 (14.0) |
| Leukemia | 29 (7.8) |
| **Treatments** |  |
| **Antibiotic use before infection (within 30 days)** |  |
| 3^rd^ Cephalosporins | 265 (71.2) |
| Carbapenems | 208 (55.9) |
| Glycopeptides | 163 (43.8) |
| Macrolides | 88 (23.7) |
| Fluoroquinolones | 30 (8.1) |
| Aminoglycosides | 43 (11.6) |
| Penicillins | 47 (12.6) |
| Tetracyclines | 14 (3.8) |
| Antifungal agents | 113 (30.4) |
| **Parenteral nutrition** | 96 (25.8) |
| **Blood transfusion (within 30 days)** | 299 (80.4) |
| **Corticosteroid therapy (within 30 days)** | 237 (63.7) |
| **Invasive operation before infection(within 30 days)** | 338 (90.9) |
| Gastric intubation | 297 (79.8) |
| Tracheal intubation | 295 (79.3) |
| Urinary catheterization | 201 (54.0) |
| Drainage intubation | 36 (9.7) |
| Central venous catheterization | 92 (24.7) |
| Bone marrow aspiration | 39 (10.5) |
| Lumbar puncture | 60 (16.1) |
| **Previous surgery (within 3 months)** | 165 (44.4) |
